# The paths of Graves’ disease in the 21st century: precision medicine is closer

**DOI:** 10.20945/2359-3997000000304

**Published:** 2020-10-08

**Affiliations:** 1 Faculdade de Ciências Médicas da Santa Casa de São Paulo Divisão de Endocrinologia Departamento de Medicina São Paulo SP Brasil Faculdade de Ciências Médicas da Santa Casa de São Paulo (FCMSCSP), Unidade de Tireoide, Divisão de Endocrinologia, Departamento de Medicina. Programa de Pós-Graduação em Ciências da Saúde, FCMSCSP, São Paulo, SP, Brasil

Graves’ disease (GD), the main cause of hyperthyroidism in areas with sufficient iodine intake ( 1 ), still poses a challenge to our scientific thinking. We seek answers about why it occurs, try to understand its pathophysiological foundations, and consider its different presentations. In the spectrum of autoimmune thyroid diseases, GD presents diverse phenotypes, clinical evolution and laboratory profile, often with complex therapeutic responses.

For decades, we have been attempting to understand the possible triggers for breaking tolerance to antigens. Likewise, we wonder which genetic susceptibility profiles are actually involved, e.g., polymorphisms of genes CTLA-4, TSHR and PTPN-22 ( 2 ). We must also understand which environmental context, in addition to smoking, plays a relevant role in the development of immune dysfunction and, consequently, thyroid dysfunction.

Graves’ disease is unique: the presence of an IgG antibody, known as TRAb, whose atypical biological effect promotes hyperfunction of the TSH receptor, more particularly, of the TSH receptor A subunit ( 3 ), not only increases thyroid hormone secretion, but also causes changes in the morphology and histology of the thyroid parenchyma, something unparalleled in autoimmune syndromes. Who would have figured that TRAb itself, in crosstalk with hyperexpressed IGF receptors in orbit fibroblasts ( 4 , 5 ), is crucial to the Graves’ orbitopathy (GO) process, occurring in 25 to 50% ( 6 ) of the cases of GD, usually in tandem with classic features of thyrotoxicosis?

In the last 30 years, we have gained a great deal of scientific knowledge about GD, determined the main predictive factors of response to pharmacological treatment, e.g., goiter volume and TRAb values, and learned how to provide better care to patients and plan their treatment. This is the consequence of new – and not so new – cumulative scientific knowledge acquired over the years. In this timeline, there have been several studies on immunoregulatory gene polymorphisms, whose role has been shown to be relevant not only for detecting the risk of development of the disease, but also as genetic predictive factors of the therapeutic and laboratory response to antithyroid drugs ( 7 ). This relevance is clear when we evaluate the HLA profile and its response to the risk of agranulocytosis, a rare but serious adverse effect of treating patients with thionamides ( 8 ). Also, much has been understood about autoimmune orbitopathy, and new drugs have been developed, ever since, to improve the quality of life of patients with severe orbital disease. One example is Teprotumumab, which has unprecedented action to block and modulate IGF receptors ( 9 ).

In this historical context, we clearly realize that euthyroidism needs to be reestablished without inducing hypothyroidism, using algorithms and mathematical models for drug dose calculation and prediction of a decrease in the levels of T4 and T3 ( 10 ) or employing unusual imaging methods (SPECT-CT) for thyroid diseases with excellent results for predicting early response to methimazole ( 11 ).

However, despite all this evolution of knowledge, the pillars of treatment still lack a more definitive agent for control of the chronic autoimmune inflammatory disease known as GD. Control of the inflammatory profile of Th2, Th1 and Th17 and the stability of the abnormal expansion of pathogenic T and B cells are seen as fundamental, because we still cannot control failures of tolerance during co-stimulation, which is crucial for an alleged cure (rather than just control) for the disease.

Thus, the treatment has remained the same for decades. Regions or countries have their preferences regarding iodotherapy, use of thionamides or even thyroidectomy ( 12 ). Currently, there is a certain tendency towards using thionamides with well-designed protocols for low-dose administration of methimazole, for a prolonged period of time, with greater remission and, therefore, less recurrence of hyperthyroidism ( 13 ).

In this sense, the research published by Pinto and cols. ( 14 ) in this edition of *Archives of Endocrinology and Metabolism* (AE&M) shows, possibly owing to a decrease in smoking in Brazil, that GD had the lowest average goiter volume in the last 30 years. This outcome, i.e., the intriguing absence of goiter at the diagnosis of GD, had already been reported by some physicians in their daily practice. The same authors also found a lower frequency of moderate and severe GO, in addition to more advanced age at diagnosis. We know that non-smoking patients with mild thyrotoxicosis, smaller goiter volume and possibly lower TRAb values have a better response to pharmacological treatment; therefore, this change found in the GD phenotype, efficiently described by the authors, justifies the priority choice of methimazole for initial and definitive treatment plan, in some cases.

Precision medicine seems to be closer to offering an accurate treatment for GD. We can plan the treatment using low doses of thionamides, control thyrotoxicosis while avoiding overdose-induced hypothyroidism by using methimazole, and have longer total treatment time without producing adverse effects on patients. We can also improve orbital inflammation without relying only on glucocorticoids. But what about controlling the autoimmune process? Another important article published in this edition of the AE&M by Saeed and cols. ( 15 ), showed that the low values of IL-27 and IL-35, of the IL-12 family (the newest marker of immune dysfunction in Graves’ disease), indicate a new path for other types of treatment for GD.

Both cytokines, IL-27 and IL-35, which appeared to have only a pro-inflammatory effect, are actually important players for control and suppression of Th1 and Th17 responses. IL-27 produces an antagonistic effect on IL-6 and regulates its anti-inflammatory effect through IL-4 and IL-10, while IL-35 has an inhibitory effect on Th1 and Th17 cells. According to the authors, such low values suggest a lesser effect of inflammation control, resulting from dysfunctional activation of self-reactive T cell clones. In the literature, recent research has associated the polymorphism (rs153109) of the IL-27 gene ( 16 ) with GD, and other researchers have corroborated these findings regarding the role of IL-35 in autoimmune thyroiditis, at different times of overt hyperthyroidism ( 17 ). Perhaps a new path for new and definitive drugs will emerge from this new point in the history of GD to restore the normality of the dysfunctional immune system.

It is very evident that the presentation of GD has changed over time and we have found, through science, good paths for treatment and therapeutic success. We can say that we know more about the cause of GD and the best path towards a more definitive control of this complex autoimmune endocrinopathy. The next steps will come in no time and the cure, therefore, does not seem to be so out of reach.
